# Postoperative stiffness requiring fibroarthrolysis does not impair long‐term outcomes after multiligament knee reconstruction

**DOI:** 10.1002/jeo2.70883

**Published:** 2026-08-06

**Authors:** Tomas Pineda, Rodrigo Olivieri, Juan Uribe, Martin Guzman, Gonzalo Idiaquez, Cristobal Diaz, Thomas Neri

**Affiliations:** ^1^ Facultad de Medicina Universidad Andrés Bello, Hospital del Trabajador Santiago Chile; ^2^ Facultad de Medicina Universidad Finis Terrae, Hospital el Carmen Santiago Chile; ^3^ Clinica Bupa Santiago Chile; ^4^ Facultad de Medicina, Universidad de Valparaiso Hospital Naval Almirante Nef Santiago Chile; ^5^ Universidad de Santiago de Chile, Facultad de Medicina Hospital del Salvador Santiago Chile; ^6^ Department of Orthopaedic and Traumatic Surgery Hospices Civils de Lyon, Hôpital Lyon Sud, Université Claude Bernard Lyon 1 Lyon France; ^7^ IFSTTAR, LBMC UMR_T9406, Gustave Eiffel University Lyon France

**Keywords:** fibroarthrolysis, functional outcomes, manipulation under anaesthesia, multiligament knee injury, postoperative stiffness

## Abstract

**Purpose:**

To evaluate whether patients requiring surgical reintervention for postoperative stiffness following multiligament knee reconstruction achieve comparable functional outcomes to those who do not require additional surgery.

**Methods:**

A retrospective cohort study was conducted including patients who underwent surgical reconstruction for multiligament knee injuries between January 2015 and February 2023 at a single level‐1 trauma centre, with a minimum follow‐up of 24 months. Patients who developed postoperative flexion stiffness requiring arthroscopic fibroarthrolysis combined with manipulation under anaesthesia were identified and compared with those who did not require reintervention. The primary outcomes were the Lysholm score and Knee injury and Osteoarthritis Outcome Score (KOOS4) score at final follow‐up.

**Results:**

A total of 95 patients were included in the final analysis, of whom 16 (16.8%) required surgical reintervention for postoperative stiffness. The mean age was 38.4 ± 13.5 years and the mean follow‐up was 73.2 ± 25.1 months. The median time from index surgery to fibroarthrolysis was 5.0 months (interquartile range, 4.0–8.3). At final follow‐up, no statistically significant differences were observed between groups in KOOS4 score (57.9 ± 2.8 vs. 62.9 ± 5.4; *p* = 0.640) or Lysholm score (64.1 ± 2.7 vs. 66.6 ± 6.0; *p* = 0.831), with minimal effect sizes (*r* = 0.05 and *r* = 0.02, respectively).

**Conclusion:**

Patients who required surgical reintervention for postoperative stiffness after multiligament knee reconstruction achieved functional outcomes comparable to those who did not require additional surgery. Arthroscopic fibroarthrolysis combined with manipulation under anaesthesia appears to be a safe and effective treatment option in this setting, supporting its consideration when conservative management has been exhausted.

**Level of Evidence:**

Level III, retrospective cohort study.

**Abbreviations**
ACL: anterior cruciate ligament
BMI: body mass index
FCL: fibular collateral ligament
IQR: interquartile range
KOOS: knee injury and osteoarthritis outcome score
MCL: medial collateral ligament
MLKI: multiligament knee injury
MUA: manipulation under anaesthesia
PCL: posterior cruciate ligament
PLC: posterolateral corner
PROMs: patient‐reported outcome measures
SD: standard deviation

## INTRODUCTION

Despite advances in surgical management, multiligament knee injuries (MLKI) continue to present with variable functional outcomes, and complications requiring reintervention remain relatively common [4, 6, 9, 11]. Among these, postoperative stiffness is one of the most frequent and clinically relevant complications, with potential implications for delayed recovery and return to activity. Several factors have been associated with its development, including the use of postoperative bracing and medial‐sided injuries, which carry a particularly elevated risk [5, 11]. Despite advances in surgical technique and fixation allowing earlier rehabilitation initiation, stiffness remains a significant challenge in this population [5, 10].

Arthroscopic fibroarthrolysis combined with manipulation under anaesthesia (MUA) is the most commonly used surgical treatment in patients with persistent stiffness [7]. However, the extent to which stiffness requiring reintervention affects long‐term functional outcomes remains unclear.

The purpose of this study was to evaluate whether patients who require surgical reintervention for postoperative stiffness after MLKI reconstruction achieve comparable functional outcomes to those who do not require additional surgery. It was hypothesised that patients undergoing reintervention would demonstrate inferior functional outcomes at final follow‐up.

## METHODS

### Patient population

**Figure 1:**
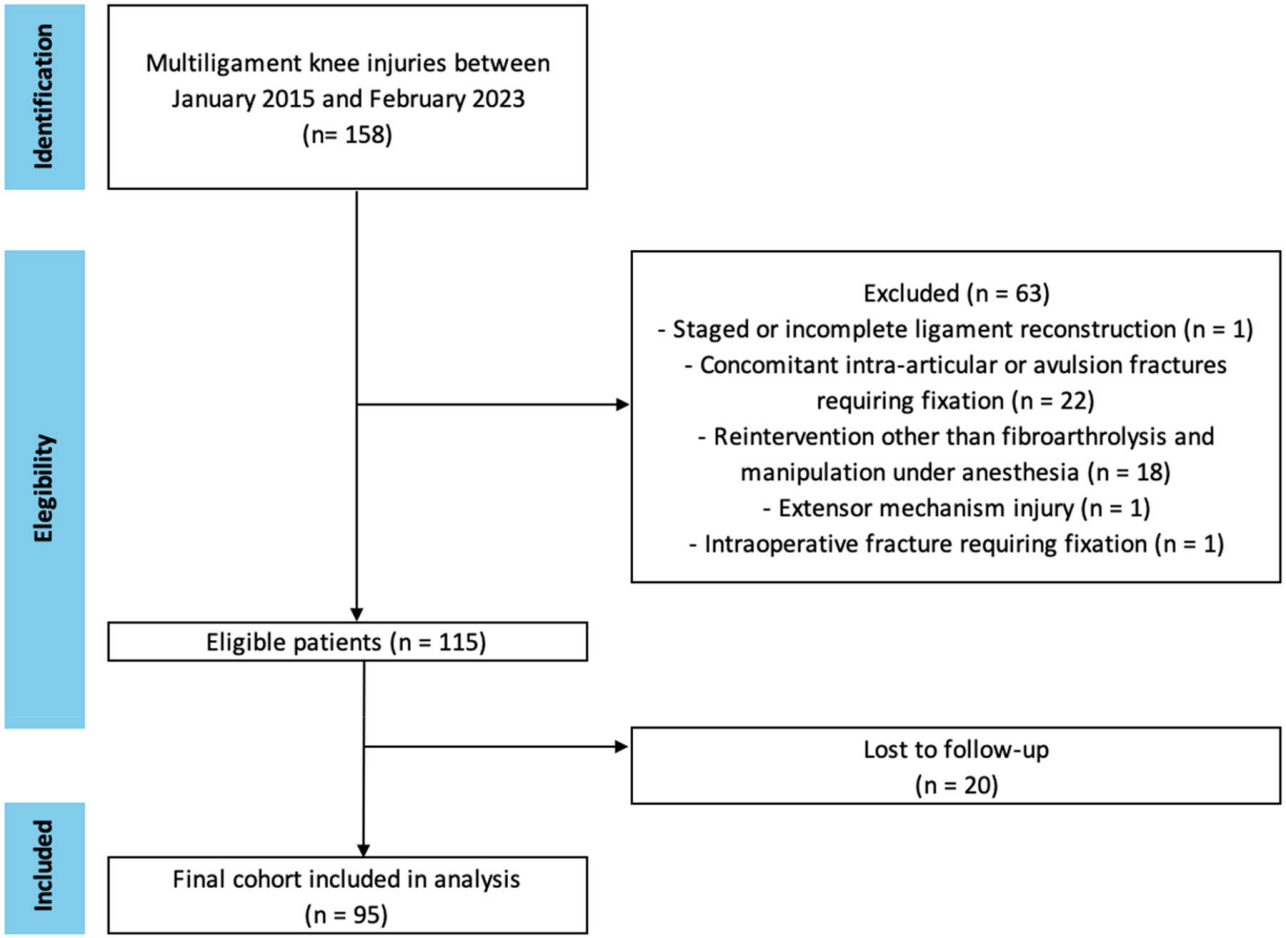
Flowchart of patients with multiligament knee injuries between January 2015 and February 2023 and the selection process of the final cohort.

A retrospective cohort study was conducted including patients undergoing surgical reconstruction for multiligament knee injuries between January 2015 and February 2023 at a single level 1 trauma centre, all treated within a workers' compensation system (Figure 1).

MLKI were defined as an injury to at least 2 of the 4 major knee ligaments of the knee requiring surgical treatment, including the anterior cruciate ligament (ACL), posterior cruciate ligament (PCL), medial collateral ligament (MCL), fibular collateral ligament (FCL) or posterolateral corner (PLC) with a minimum 24‐month follow‐up.

Inclusion criteria were patients aged 18 years or older who underwent surgical treatment for purely ligamentous multiligament knee injuries within the study period.

Patients with concomitant intra‐articular knee fractures or avulsion fractures requiring surgical treatment were excluded, as were those who underwent revision ligament reconstruction, concomitant osteotomy, or had extensor mechanism injuries. Patients requiring reinterventions other than fibroarthrolysis combined with MUA prior to final follow‐up were also excluded.

Patients requiring surgical intervention for postoperative flexion deficit were identified within the cohort and compared with those who did not require additional procedures. Stiffness was defined as a flexion deficit below 90° persisting despite supervised rehabilitation for at least 3 months. Arthroscopic fibroarthrolysis combined with MUA was indicated for all patients who met this criterion. In patients with flexion between 90° and 100° but with functionally limiting symptoms, the decision to proceed with surgical management was made jointly by the treating surgeon and the patient after individualised assessment. The latter patients were included in the reintervention group for the purposes of this analysis.

### Baseline characteristics

Patient charts were retrospectively reviewed to obtain baseline characteristics. Age at the time of injury, sex, date of injury, side of injury, mode of injury, type of injury, meniscal injuries, surgical procedures, reintervention and complications were obtained.

A classification system based on the pattern was utilised as follows: Type I: ACL or PCL and MCL or FCL, Type II: ACL and PCL, Type III: ACL, PCL and FCL or MCL and Type IV: ACL, PCL, FCL and MCL. A high‐energy mechanism was defined as motor vehicle accidents, falls from heights more than 2 m (6.5 feet), otherwise were considered low‐energy mechanisms.

### Patient follow‐up and patient‐reported outcome measures (PROMs)

At a minimum follow‐up of 24 months, patients were contacted by telephone and email and queried regarding any reintervention. Patients were considered lost to follow‐up if no response was obtained after five telephone calls and three emails over a period of 6 months. The knee‐specific PROMs included Lysholm score and KOOS4 score.

### Surgical technique

All reconstructions were performed in a single‐stage procedure using anatomic techniques. During this period of time, the specific surgical approach, including graft selection and reconstruction strategy, was determined at the discretion of the treating surgeon.

The surgical treatment of postoperative stiffness consisted of arthroscopic fibroarthrolysis performed through standard anteromedial and anterolateral portals. A systematic diagnostic arthroscopy was performed at the outset of each procedure to assess the extent and distribution of fibrotic tissue, identify any associated intra‐articular pathology, and guide the release strategy. Fibrous adhesions were resected using a combination of mechanical shavers and radiofrequency devices, targeting the suprapatellar pouch, medial and lateral gutters and the intercondylar notch as needed. Posteromedial and posterolateral portals were added when necessary at the discretion of the surgeon to address posterior capsular adhesions and restore terminal extension. Once complete arthroscopic release was confirmed, MUA was performed at the end of the procedure, with the goal of achieving full knee extension and at least 90° of flexion intraoperatively. No patient required open arthrolysis.

### Postoperative and rehabilitation protocol

Following multiligament reconstruction, patients followed a structured rehabilitation protocol including early quadriceps activation and protected weight‐bearing with crutches. A knee brace was used in all cases; in patients with posterior cruciate ligament involvement, a PCL‐specific dynamic knee orthosis (M.4s PCL dynamic, medi GmbH & Co. KG) was applied to maintain anterior tibial translation and allow early controlled range of motion. Range of motion was initiated within the first postoperative days, targeting 0°–90° of flexion during the first four weeks, with progressive advancement thereafter. Weight‐bearing was initially restricted to partial load and subsequently progressed to full weight‐bearing as tolerated, typically by weeks 6–8. Closed‐chain strengthening exercises were introduced once an adequate range of motion was achieved, with open‐chain quadriceps exercises initiated in the early postoperative period. In patients with PCL reconstruction, open‐chain hamstring strengthening was avoided during the first 12 weeks to prevent posterior tibial translation. Strengthening and functional exercises were progressively introduced as range of motion and neuromuscular control improved.

After arthroscopic fibroarthrolysis combined with MUA, patients remained hospitalised for 2 days and initiated rehabilitation on the day of surgery, including the use of continuous passive motion. Weight‐bearing was allowed as tolerated. Patients followed a structured physiotherapy program consisting of supervised sessions five times per week, complemented by daily home‐based exercises. Rehabilitation focused on maintaining the achieved range of motion and progressing toward functional recovery.

### Statistical analysis

Continuous variables were assessed for normality using the Shapiro–Wilk test and visual inspection of histograms and Q–Q plots. Data are presented as mean ± standard deviation or median with interquartile range, according to distribution, while categorical variables are reported as frequencies and percentages.

Comparisons between patients who required reintervention for stiffness and those who did not were performed using independent samples *t*‐tests or Mann–Whitney *U* tests, as appropriate. Categorical variables were compared using the chi‐square test or Fisher's exact test. Effect sizes were calculated to estimate the magnitude of differences between groups.

A *p*‐value < 0.05 was considered statistically significant. Statistical analysis was performed using SPSS version 27 (IBM Corp.).

## RESULTS

### Patient characteristics

A total of 95 patients were included in the final analysis, after exclusion of 20 patients (17.5%) lost to follow‐up, of whom 16 (16.8%) required surgical reintervention for postoperative stiffness. The mean age at the time of injury was 38.4 ± 13.5 years and the mean follow‐up was 73.2 ± 25.1 months. In total, 60 patients (63.2%) had high‐energy injuries, and 35 (36.8%) had low‐energy injuries (Table 1).

**Table 1:** Demographic and clinical characteristics of the study population.

Variable	Total (*n* = 95)	No stiffness group (*n* = 79)	Stiffness requiring surgery (*n* = 16)	*p*‐value
Age (years), mean ± SD	38.4 ± 13.5	37.9 ± 13.6	40.5 ± 13.3	0.412
BMI (kg/m^2^), mean ± SD	29.6 ± 5.5	29.7 ± 5.8	29.3 ± 4.1	0.800
Male, *n* (%)	63 (66.3%)	51 (64.6%)	12 (75.0%)	0.692
High energy, n (%)	60 (63.2%)	48 (60.8%)	12 (75.0%)	0.255
Follow‐up (months), mean ± SD	73.2 ± 25.1	74.6 ± 25.3	66.2 ± 24.2	0.231

*Note*: Continuous variables are presented as mean ± SD or median (range), and categorical variables as number (%). Statistical significance was defined as *p* < 0.05.

Abbreviations: BMI, body mass index; SD, standard deviation.

Concurrent meniscal procedures were performed in a substantial proportion of patients. Meniscal repair was performed in 30 patients (31.6%), including 11 (11.6%) medial and 11 (11.6%) lateral repairs. Partial meniscectomy was performed in 22 patients (23.2%), including 13 (13.7%) medial and 17 (17.9%) lateral procedures. Medial‐sided injuries were present in 48 patients (50.5%) overall, including 39 (49.4%) in the no‐stiffness group and 9 (56.2%) in the stiffness group, with no statistically significant difference between groups (*p* = 0.785). Reconstruction of three or more ligaments was performed in 7 of 16 patients (43.8%) in the stiffness group versus 19 of 79 (24.1%) in the no‐stiffness group (*p* = 0.192). The proportion of KD‐I injuries did not differ significantly between groups (62.5% vs. 70.9%; *p* = 0.714). The distribution of multiligament injury patterns and associated lesions is presented in Table 2.

**Table 2:** Injury characteristics and associated lesions according to MLKI pattern.

Multiligament injury pattern	*n* (%)	High energy	Meniscal injury	Nerve injury	Vascular injury	Stiffness requiring surgery
MLKI‐I	66 (69.5%)	61 (64.2%)	26 (27.4%)	2 (2.1%)	1 (1.1%)	10 (15.2%)
MLKI‐II	6 (6.3%)	6 (6.3%)	3 (3.2%)	0	1 (1.1%)	0
MLKI‐III	14 (14.7%)	13 (13.7%)	7 (7.4%)	0	0	4 (4.2%)
MLKI‐IV	9 (9.5%)	9 (9.5%)	4 (4.2%)	2 (2.1%)	1 (1.1%)	2 (22.2%)
Total, *n* (%)	95 (100%)	89 (93.7%)	40 (42.1%)	4 (4.2%)	3 (3.2%)	16 (16.8%)

*Note*: Values are presented as number (percentage). Percentages are calculated based on the total study population.

Abbreviation: MLKI, multiligament knee injury.

The median time from index surgery to fibroarthrolysis was 5.0 months (IQR, 4.0–8.3).

At final follow‐up, no statistically significant differences were observed between patients who required reintervention and those who did not. The KOOS4 score was comparable between groups (57.9 ± 2.8 vs. 62.9 ± 5.4; *p* = 0.640, 95% CI: −7.0 to 17.0), as was the Lysholm score (64.1 ± 2.7 vs. 66.6 ± 6.0; *p* = 0.831, 95% CI: −10.6 to 15.5), with negligible effect sizes between groups (*r* = 0.05 for KOOS4 and 0.02 for Lysholm). Descriptive functional outcomes stratified by injury pattern are presented in Table 3.

**Table 3:** Descriptive functional outcomes by MLKI pattern.

MLKI pattern	*n*	KOOS4	Lysholm
MLKI‐I	66	61.4 (36.7–80.9)	75.5 (41.0–86.0)
MLKI‐II	6	64.1 (57.3–69.0)	63.0 (58.2–67.8)
MLKI‐III	14	63.3 (47.1–83.0)	74.5 (57.2–80.0)
MLKI‐IV	9	52.3 (40.8–68.2)	68.0 (56.0–72.0)

*Note*: Values are presented as median (interquartile range).

Abbreviations: KOOS, knee injury and osteoarthritis outcome score; MLKI, multiligament knee injury.

No patient in the reintervention group required a subsequent surgical procedure for recurrent stiffness during the follow‐up period, and no complications were recorded following arthroscopic fibroarthrolysis.

## DISCUSSION

The most important finding of the present study is that patients who required surgical reintervention for postoperative stiffness following multiligament knee reconstruction achieved functional outcomes at final follow‐up comparable to those of patients who did not require additional surgery, when stiffness was diagnosed and treated at an early stage.

Postoperative stiffness has been consistently reported as one of the most frequent complications following MLKI reconstruction, with a subset of patients requiring additional surgical management [4, 5]. However, the impact of this complication on final functional outcomes remains insufficiently characterised. Previous literature has primarily focused on complication rates rather than on the functional consequences of stiffness requiring surgical treatment [1, 11]. In this regard, Fahlbusch et al. [3] conducted a systematic review of 25 studies specifically addressing arthrofibrosis following MLKI reconstruction, reporting it as a common but poorly defined complication, with the majority of included studies employing imprecise and subjective definitions. This methodological heterogeneity limits direct comparisons across the literature and should be considered when interpreting reintervention rates across studies.

The risk of developing postoperative stiffness is not uniform across this population. Early surgery, reconstruction of three or more ligaments and medial‐sided injuries have been identified as primary risk factors in the existing literature [4, 11, 12]. In the present cohort, a substantial proportion of patients had three or more ligaments reconstructed, which may partly account for the observed reintervention rate, placing it in the upper range of figures reported in the literature. Importantly, this complication rate did not translate into inferior functional outcomes at final follow‐up, suggesting that when stiffness is identified and appropriately managed, its impact on the overall recovery trajectory may be limited.

In parallel, recent studies evaluating patient‐reported outcomes after MLKI have demonstrated that functional recovery remains variable and is influenced by multiple factors, including injury pattern and treatment strategy [2, 6, 7, 14]. Within this context, the present findings suggest that postoperative stiffness requiring reintervention, when appropriately managed, may not be a dominant determinant of long‐term functional outcome. In addition, the very small effect sizes observed suggest that group differences were minimal, reinforcing the absence of clinically meaningful differences between groups.

The timing of arthroscopic fibroarthrolysis in this cohort warrants consideration. The median interval from index surgery to fibroarthrolysis was 5 months. Existing evidence suggests that earlier intervention, once adequate maturation of scar tissue has occurred and conservative rehabilitation has been exhausted, may be associated with more favourable range‐of‐motion recovery, although the optimal timing window remains a subject of debate [13]. These findings are further supported by Lamba et al. [8], who reported satisfactory patient‐reported outcomes and high patient satisfaction at a mean follow‐up of 7 years following arthroscopic lysis of adhesions and MUA for arthrofibrosis after MLKI reconstruction. While functional scores were broadly consistent with those observed in the reintervention group of the present cohort, the present study extends these findings to a larger sample and provides a direct comparison with a concurrent non‐stiffness group, demonstrating that reintervention does not adversely affect long‐term functional outcomes relative to patients with an uncomplicated postoperative course. In the present study, all patients completed a minimum of 3 months of supervised rehabilitation prior to surgical indication, which may have contributed to the favourable outcomes observed in the reintervention group. The absence of patients requiring open arthrolysis further supports the effectiveness of the arthroscopic approach when applied within an appropriate timeframe.

From a clinical perspective, these findings are relevant because the need for reintervention is frequently interpreted as a marker of an unfavourable postoperative course [4]. However, the present results suggest that when stiffness is identified early and addressed promptly, patients may achieve functional outcomes comparable to those without this complication. This information may be useful for patient counselling and postoperative decision‐making, reinforcing the importance of early recognition and prompt surgical management of postoperative stiffness in this population.

A secondary observation of relevance is that both groups demonstrated moderate functional scores at final follow‐up. This is consistent with the trajectory described in recent meta‐analytic data, which show progressive functional decline over time following MLKI reconstruction, a pattern that appears particularly pronounced in PCL‐based injuries [6]. The scores observed in the present cohort, obtained at a mean follow‐up exceeding 6 years, are in keeping with this deterioration trend and are likely reflective of the natural history of these complex injuries rather than a consequence of stiffness or its surgical management.

Several limitations should be acknowledged. The retrospective design introduces potential sources of bias. The study was conducted exclusively within a workers' compensation system, a context known to be associated with lower patient‐reported outcome scores independent of injury severity, which may limit generalisability to non‐compensation populations. The indication for reintervention was not standardised and was based on surgeon judgement, however, all patients underwent a minimum of 3 months of rehabilitation prior to surgical indication, which partially mitigates this limitation. In addition, the retrospective nature of the study precluded the systematic capture of objective ROM thresholds at the time of surgical indication, and the criteria applied likely reflect individualised clinical decision‐making rather than a strict protocol. The cohort encompasses a heterogeneous spectrum of injury patterns, with variable ligament combinations and reconstruction strategies, including graft selection, which were left to the discretion of the treating surgeon. This variability introduces confounding that may independently influence both the risk of postoperative stiffness and functional outcomes, and should be considered when interpreting the present findings. The primary limitation of this study is its limited statistical power. With only 16 patients in the reintervention group, the study was underpowered to detect small or moderate between‐group differences, substantially increasing the risk of type II error. This sample size also precluded subgroup or multivariable adjusted analyses that would have been necessary to account for the aforementioned heterogeneity. Finally, the absence of baseline functional scores precludes assessment of preoperative comparability in terms of patient‐reported outcomes.

## CONCLUSION

Patients who required surgical reintervention for postoperative stiffness after multiligament knee reconstruction achieved functional outcomes comparable to those who did not require additional surgery. Arthroscopic fibroarthrolysis combined with MUA appears to be a safe and effective treatment option in this setting, supporting its consideration when conservative management has been exhausted.

## AUTHOR CONTRIBUTIONS

**Martin Guzman**: Data curation; writing—review and editing. **Cristobal Diaz**: Data curation; writing—review and editing. **Gonzalo Idiaquez**: Data curation; writing—review and editing. **Juan Cristobal Uribe**: Formal analysis; writing original draft. **Tomas Pineda**: Formal analysis; writing original draft. **Thomas Neri**: Supervision; methodology. **Rodrigo Olivieri**: Supervision; methodology.

## FUNDING INFORMATION

The authors have no funding to report.

## CONFLICT OF INTEREST STATEMENT

The authors have nothing to report.

## ETHICS STATEMENT

This study was approved by the Institutional Review Board of Hospital del Trabajador (Ethics Committee approval number: CEC/04/2025). The study was conducted in accordance with the Declaration of Helsinki.

## Data Availability

The data that support the findings of this study are available on request from the corresponding author. The data are not publicly available due to privacy or ethical restrictions.
